# Antibody Light Chains Dictate the Specificity of Contact Hypersensitivity Effector Cell Suppression Mediated by Exosomes

**DOI:** 10.3390/ijms19092656

**Published:** 2018-09-07

**Authors:** Katarzyna Nazimek, Philip W. Askenase, Krzysztof Bryniarski

**Affiliations:** 1 Department of Immunology, Jagiellonian University Medical College, 31-121 Krakow, Poland; katarzyna.nazimek@uj.edu.pl; 2 Section of Rheumatology, Allergy and Clinical Immunology, Yale University School of Medicine, New Haven, CT 06520, USA; philip.askenase@yale.edu

**Keywords:** antibody light chains, cell–cell signaling, immune regulation, immune suppression, exosomes, extracellular vesicles, miRNA

## Abstract

Antibody light chains (LCs), formerly considered a waste product of immunoglobulin synthesis, are currently recognized as important players in the activation of the immune response. However, very little is known about the possible immune regulatory functions of LCs. Recently, we reported that hapten-specific LCs coat miRNA-150-carrying exosomes produced by CD8+ suppressor T cells downregulating the contact hypersensitivity (CHS) reaction in an antigen-specific manner, in mice tolerized by intravenous administration of a high dose of hapten-coupled syngeneic erythrocytes. Thus, the current studies aimed at investigating the role of hapten-specific LCs in antigen-specific, exosome-mediated suppression of CHS effector cells. Suppressor T cell-derived exosomes from tolerized B-cell-deficient µMT^−/−^, NKT-cell-deficient Jα18^−/−^, and immunoglobulin-deficient JH^−/−^ mice were nonsuppressive, unless supplemented with LCs of specificity strictly respective to the hapten used for sensitization and CHS elicitation in mice. Thus, these observations demonstrate that B1-cell-derived LCs, coating exosomes in vivo and in vitro, actually ensure the specificity of CHS suppression. Our research findings substantially expand current understanding of the newly discovered, suppressor T cell-dependent tolerance mechanism by uncovering the function of antigen-specific LCs in exosome-mediated, cell–cell communication. This express great translational potential in designing nanocarriers for specific targeting of desired cells.

## 1. Introduction

Freely circulating antibody light chains (LCs) were formerly considered a waste product of immunoglobulin synthesis by B lymphocytes. However, currently the biological functions of LCs are being recognized. Due to their involvement in pathogenesis of various diseases, LCs are now considered as possible diagnostic markers [1,2,3]. Further, LCs play an important role in activation of mast cells and neutrophils, as well as in humoral immunity by supporting the antigen binding with sufficient affinity by specific antibodies [1]. This is often associated with the development of various chronic inflammatory disorders and tumor-related inflammation, in which LCs seem to promote the pathological immune reactivity of particular cells [1,4,5]. On the other hand, very little is known about the possible beneficial immune regulatory function of antibody LCs.

In contrast to B2 population, B1 lymphocytes are activated in a T cell-independent manner, by hapten-self-protein complexes and IL-4 released by hepatic natural killer T (NKT) cells stimulated with endogenous glycolipids [6,7,8,9,10,11]. During the humoral immune response B2 lymphocytes generate highly antigen-specific antibodies of various classes, depending on the route of administration of T-dependent antigen and its character. Whereas, B1 lymphocytes mostly produce natural IgM antibodies characterized by low specificity to T-independent antigen due to the lack of signals from helper T cells that could promote B cell maturation, immunoglobulin class switching, and generation of memory cells [6]. However, B1 lymphocytes are involved in an early phase of elicitation of contact hypersensitivity (CHS) reaction by delivering IgM antibodies and their LCs that bind haptenized antigens to further activate complement. This results in activation of local inflammation, predominantly mediated by mast cells and neutrophils, which finally allows for migration and recruitment of antigen-specific effector T cells to the site of hapten deposition to elicit the local symptoms of CHS reaction [6,7,8,9,10,11]. Interestingly, activation of innate-like B cells, likely resembling B1 lymphocytes, but expressing regulatory phenotype, by mouse invariant NKT cells was recently reported [12]. This suggests that B1 cells may also play a role in immune regulation. Furthermore, one of the B1a cell subsets was shown to produce IgM antibodies and LCs that express high affinity to antigen due to immunoglobulin V-region mutations caused by activation-induced cytidine deaminase (AID) [6]. These B1a cells occurred to mediate an early, protective immune response to pneumococci in a mouse model of pneumococcal pneumonia [13].

Formerly, it was reported that intravenous administration of a high dose of syngeneic mouse red blood cells (MRBCs) coupled with hapten prior to contact sensitization with the same hapten leads to the suppression of CHS reaction in mice [14]. Then, it occurred that the delivery of haptenized MRBCs induces CD8+ suppressor T (Ts) lymphocytes responsible for downregulation of CHS reaction [14,15]. Recently, we have shown that Ts cells, when activated by intravenous administration of a high dose of syngeneic MRBCs coupled with hapten, release miRNA-150-carrying exosomes that actually suppress CHS reaction induced with the same hapten [16,17,18]. After detailed characterization of Ts cell exosomes [16] (Figure S1), by employing miRNA sequencing, antimiRs and finally miRNA-150^−/−^ mice, we proved that miRNA-150 is a crucial regulatory molecule derived by the exosomes [16] to antigen-presenting cells finally suppressing CHS effector T lymphocytes [18]. Experiments with non-cross-reacting haptens; i.e., trinitrophenol (TNP) and oxazolone (OX), proved that Ts cell-derived exosomes suppress CHS reaction in an antigen-specific manner [16,17]. This surprising observation further led to the discovery that Ts cell-derived exosomes are coated with antibody LCs, as demonstrated by the means of flow cytometry (Figure S1 and Figure 5B of a past paper [16]). Afterwards, the experiments with pan immunoglobulin JH^−/−^ mice demonstrated that the surface coating of Ts cell-derived exosomes with hapten-specific LCs is essential for ensuring their suppressive activity in CHS [16]. Conversely, JH^−/−^ mouse Ts cell exosomes in vitro inhibited the IL-2-dependent viability of HT-2 T cells in a miRNA-150-dependent manner, which confirmed that they carry miRNA-150 and are only lacking LCs on their surface [16]. Additionally, coating with hapten-specific LCs enabled exosome separation by antigen affinity chromatography [16] and was suspected to provide the ability of Ts cell exosomes to target hapten-primed antigen-presenting cells that ultimately inhibit CHS effector T cells [18,19]. Therefore, we assumed that LCs in fact could be responsible for antigen-specificity of CHS suppression mediated by Ts cell-derived exosomes, however it remained a matter of speculation. Since defining the beneficial immune regulatory function of free LCs would not only allow us to fully understand the exosome-mediated cellular interactions and would also favor the recognition of the biological importance of LC, we therefore have undertaken the current studies to investigate the role of hapten-specific LCs in an antigen-specific, Ts cell-exosome-mediated suppression of CHS effector cells.

## 2. Results

### 2.1. B Cells Deliver Hapten-Specific LCs for Coating of Ts Cell-Derived Exosomes to Enable Their Suppressive Function

**Figure 1:**
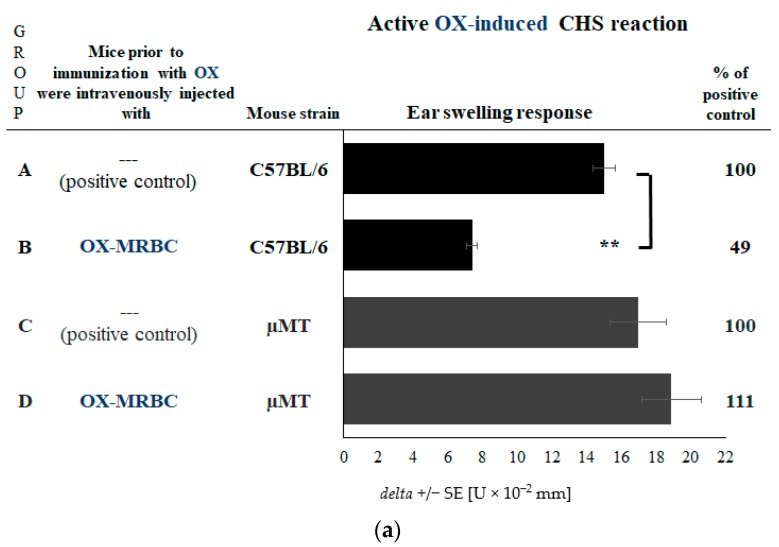

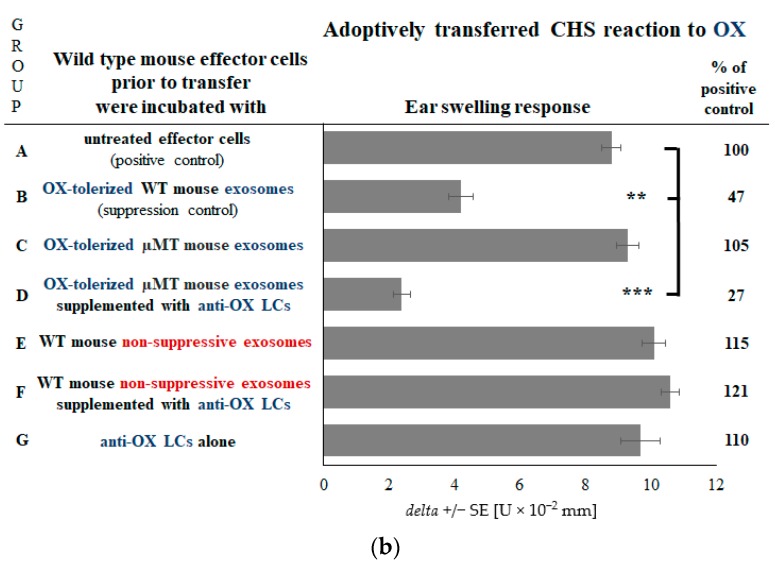
Effects of intravenous administration of a high dose of oxazolone (OX)-coupled syngeneic mouse red blood cells (MRBC) to wild type (C57BL/6) or B cell-deficient µMT mice on contact hypersensitivity (CHS) reaction. CHS reaction was measured as ear swelling response either (**a**) in actively sensitized mice that had been administered with OX-MRBC or (**b**) in recipients of CHS effector cells incubated with exosomes (in some instances supplemented with anti-OX antibody light chains—LCs) generated by lymph node and spleen T suppressor cells of mice injected with OX-MRBC. Bars express delta ± standard error (SE). *n* = 5 mice in each group. ** *p* < 0.01; *** *p* < 0.001.

We have previously observed that coating of Ts cell-derived exosomes with hapten-specific LCs is required to accomplish exosome suppressive function [16]. However, it was not clear if administration of haptenized MRBCs activates B lymphocytes along with Ts cells, and whether they are necessary for generation of regulatory exosomes by Ts cells. To test these assumptions, we have administered syngeneic MRBCs coupled with OX hapten into wild type (WT) and B cell-deficient µMT mice prior to their contact sensitization with OX and elicitation of CHS ear swelling response. While injection of haptenized sMRBC to WT mice led to significant suppression of CHS, it failed to inhibit the CHS reaction in B cell-deficient mice (Figure 1a, group D vs. C and B vs. A). This implied that the suppression of CHS cannot be developed in the absence of B cells. To examine if B cells play a role already at the time of exosome generation or, instead, they enable exosomes to accomplish their function, we have collected lymph nodes and spleens of hapten-coupled-MRBC-tolerized µMT mice and processed their single-cell-suspensions for Ts cell exosome isolation [16,17,18]. The yielding µMT mouse exosomes were then used to treat adoptively transferred CHS effector cells, but they failed to suppress elicited CHS reaction (Figure 1b, group C vs. A and B). Remarkably, supplementation of µMT mouse Ts cell exosomes with OX-specific LCs restored their suppressive activity (Figure 1b, group D vs. A), while addition of these LCs to initially nonsuppressive, control nanovesicles [16,17,18] had no inhibitory effect (Figure 1b, groups E and F vs. A). These observations allowed us to conclude that B cells are not necessary for generation of suppressive exosomes by Ts cells, but, instead, they deliver hapten-specific LCs for coating of exosomes, which is crucial to accomplish their function. Further, as LCs alone, or when coating the nonsuppressive nanovesicles, were noninhibitory, we considered that they solely enable execution of suppression by Ts cell exosomes.

### 2.2. CHS Reaction Is Not Suppressed in the Absence of NKT Cells

**Figure 2:**
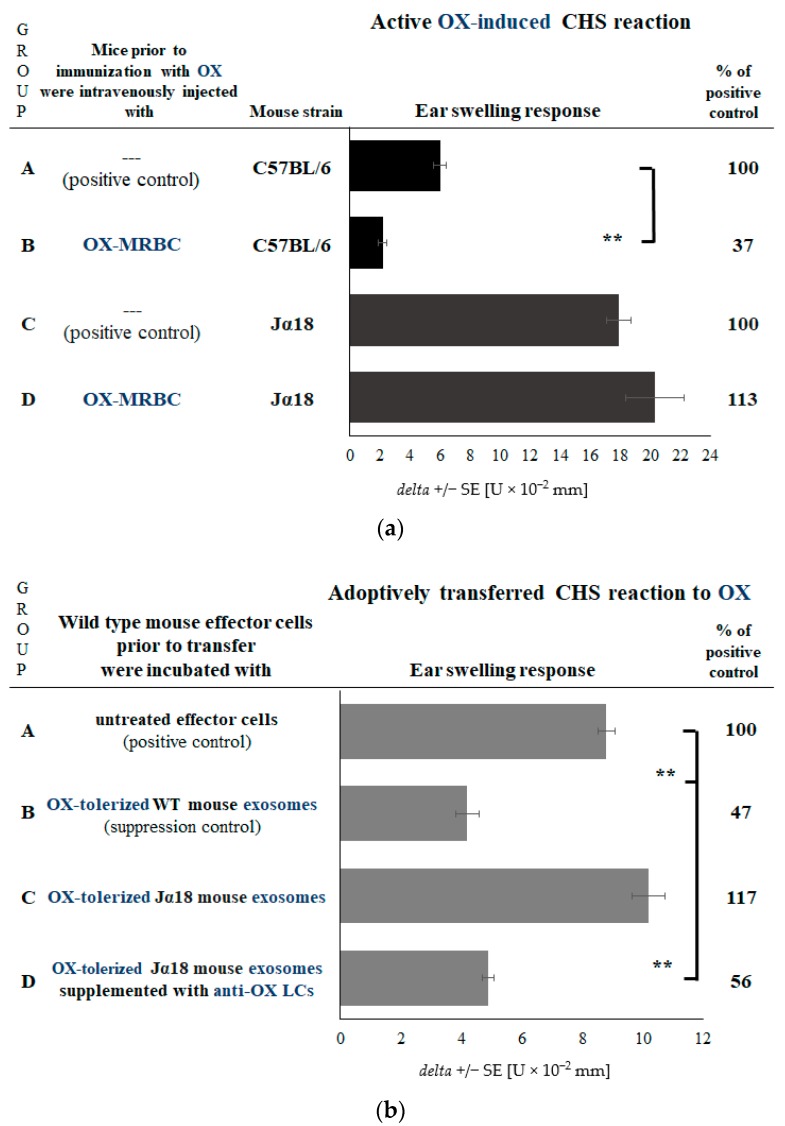
Effects of intravenous administration of a high dose of oxazolone (OX)-coupled syngeneic mouse red blood cells (MRBC) to wild type (C57BL/6) or NKT cell-deficient Jα18 mice on contact hypersensitivity (CHS) reaction. CHS reaction was measured as ear swelling response either (**a**) in actively sensitized mice that had been administered with OX-MRBC or (**b**) in recipients of CHS effector cells incubated with exosomes (in some instances supplemented with anti-OX antibody light chains—LCs) generated by lymph node and spleen T suppressor cells of mice injected with OX-MRBC. Bars express delta ± standard error (SE). *n* = 5 mice in each group. ** *p* < 0.01.

According to previous findings, NKT cell-activated B1 lymphocytes [6,7,8,9,10] and their derived LCs are involved in the development of an early phase of CHS effector response [6,20,21]. Thus, we speculated that the B1 cell subpopulation may also be responsible for the delivery of hapten-specific LCs then coating suppressive exosomes. We have initially verified this hypothesis by employing Jα18 mice that lack NKT cells, but have conventional T cells [22], so thus are characterized by impaired activation of B1 cells [8]. Tolerization of Jα18 mice with haptenized MRBCs did not cause the suppression of CHS reaction (Figure 2a, group D vs. C). However, the generation of Ts cell exosomes stimulated by hapten-coupled MRBCs was preserved in Jα18 mice, and the suppressive activity of these exosomes was again restored by supplementation with hapten-specific LCs (Figure 2b, group D vs. A and C). This implied that, indeed, B1 lymphocytes activated by NKT cells at the time of contact sensitization with hapten [6,7,8,9,10] are the source of hapten-specific LCs for coating of Ts cell-derived exosomes.

### 2.3. CHS Reaction Is Suppressed by Exosomes in Mice Tolerized with Syngeneic MRBCs Regardless of Their Hapten Coupling

**Figure 3:**
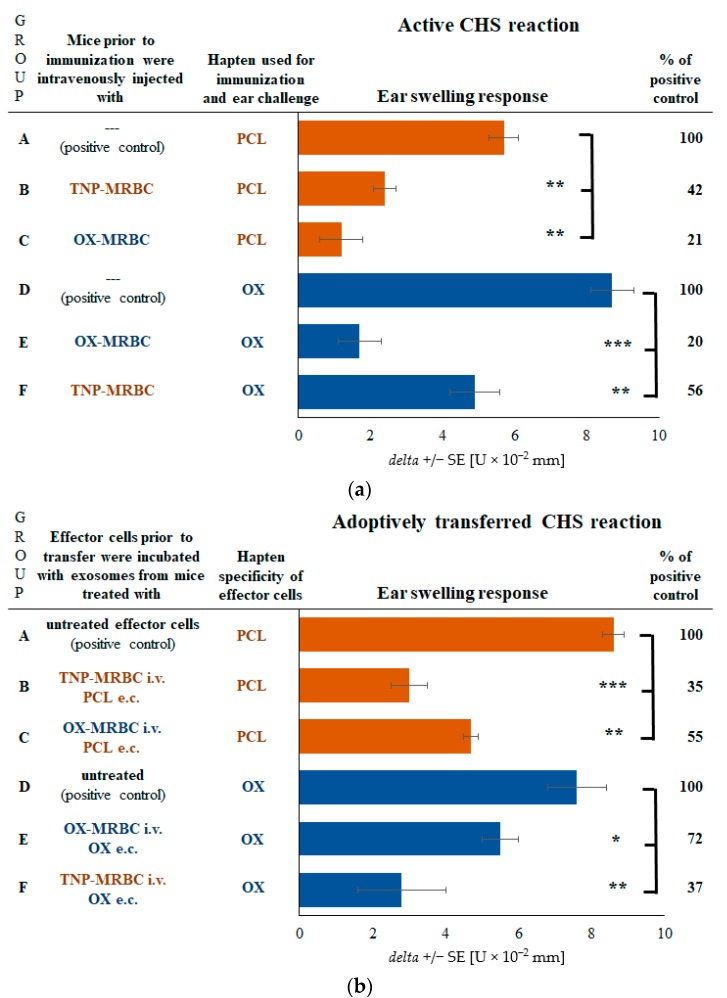
Effects of intravenous administration to CBA mice of a high dose of either trinitrophenol (TNP) or oxazolone (OX)-coupled syngeneic mouse red blood cells (MRBC) on contact hypersensitivity (CHS) reaction to TNP-chloride (PCL) or OX hapten. CHS reaction was measured as ear swelling response either (**a**) in actively sensitized mice that had been administered with hapten-coupled MRBC or (**b**) in recipients of CHS effector cells incubated with exosomes generated by lymph node and spleen T suppressor cells of mice either injected with TNP-MRBC and sensitized with PCL, injected with OX-MRBC and sensitized with PCL, injected with OX-MRBC and sensitized with OX, or injected with TNP-MRBC and sensitized with OX. Bars express delta ± standard error (SE). *n* = 5 mice in each group. * *p* < 0.05; ** *p* < 0.01; *** *p* < 0.005.

According to the current findings, we assumed that B1 cells delivering LCs are activated at the time of contact sensitization, but not under the influence of intravenously administered MRBCs coupled with hapten. Then, the suppression of CHS should be accomplished regardless of the hapten coupling of MRBCs. Thus, we have administered mice with either TNP-MRBCs or OX-MRBCs prior to contact sensitization with either PCL or OX. In all cases elicited CHS ear swelling was significantly inhibited (Figure 3a, groups B and C vs. A, and E and F vs. D). Further, we have collected lymph node and spleen cells of those mice and processed them as for Ts cell exosome harvesting. Yielded exosomes were used to treat adoptively transferred TNP-specific or OX-specific CHS effector cells, which led to significant suppression of elicited CHS reaction (Figure 3b, groups B and C vs. A, and E and F vs. D). This allowed us to conclude that haptenized MRBC administration induces Ts cells to release exosomes that gain, after contact sensitization, the surface coating with LCs of specificity dictated by sensitizing hapten, activating B1 cells.

### 2.4. CHS Reaction to TNP Hapten Is Suppressed in Mice Administered with Non-Self-Protein-Coupled Syngeneic MRBCs

**Figure 4:**
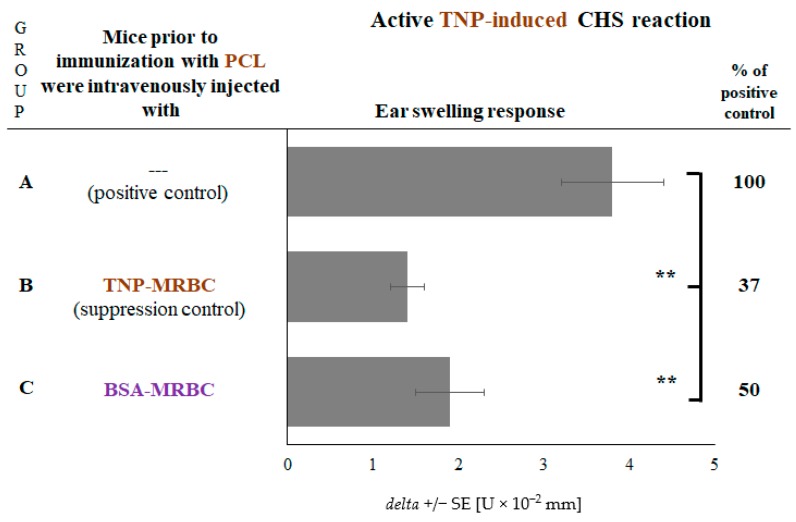
Effects of intravenous administration to CBA mice of a high dose of bovine serum albumin (BSA)-coupled syngeneic mouse red blood cells (MRBC) on contact hypersensitivity (CHS) reaction to TNP-chloride (PCL) or OX hapten. CHS reaction was measured as ear swelling response in actively sensitized mice that had been administered with BSA-MRBC prior to sensitization with PCL. Bars express delta ± standard error (SE). *n* = 5 mice in each group. ** *p* < 0.01.

Afterwards, we attempted to assess whether the hapten-coupling of syngeneic MRBCs somehow supports or restricts the subsequent ability of Ts cell exosomes to be coated with hapten-specific LCs. For this purpose, we have administered mice with either TNP-coupled or bovine serum albumin (BSA)-coupled MRBCs prior to contact sensitization with PCL, which in both cases led to significant suppression of elicited CHS reaction (Figure 4, groups B and C vs. A). Thus, we concluded that administration of altered-self MRBCs is sufficient to induce Ts cells to release suppressive exosomes that become functionally active when coated with LCs of specificity ultimately dictated by sensitizing antigen.

### 2.5. Coating of Exosomes with LCs Ensures the Antigen-Specificity of Suppression of CHS Effector Cells

**Figure 5:**
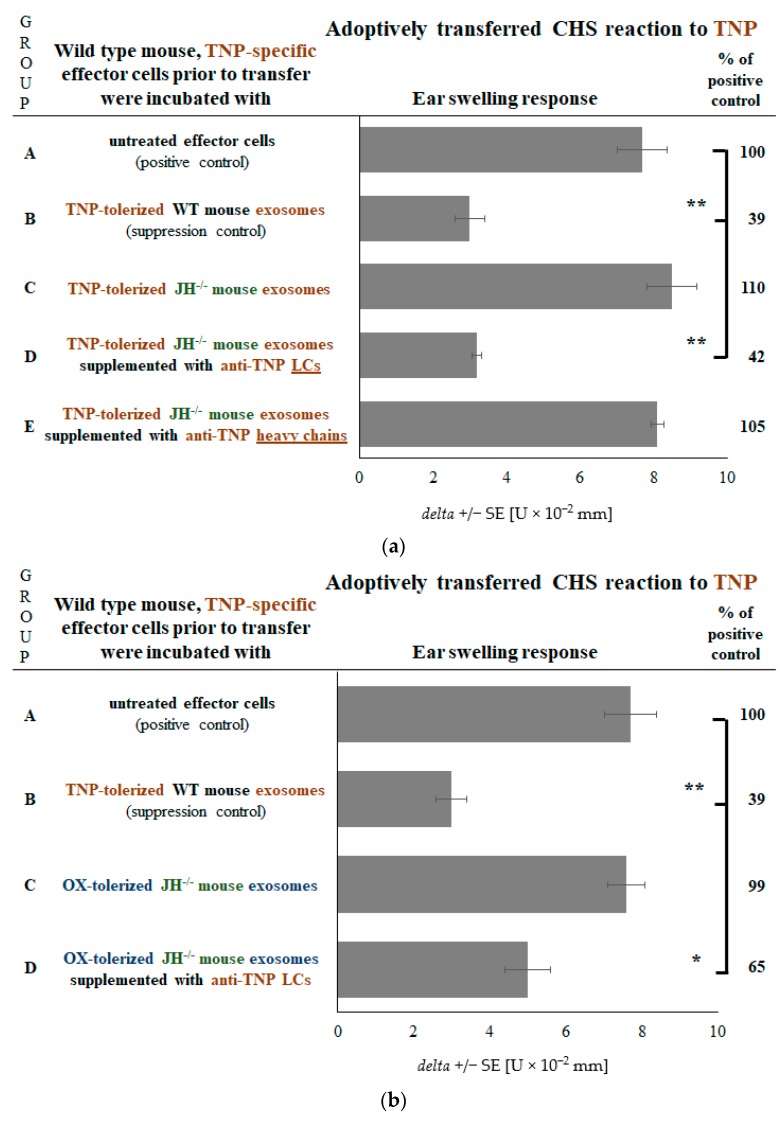

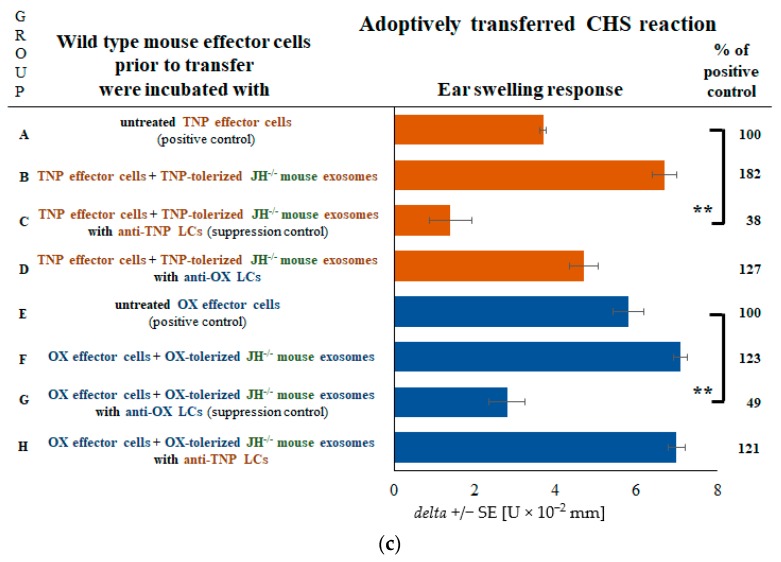
Effects of supplementation of exosomes from pan immunoglobulin-deficient JH^−/−^ mice (of BALB/c background) tolerized with either oxazolone (OX) or trinitrophenol (TNP)-coupled syngeneic mouse red blood cells (MRBC) with anti-OX or anti-TNP antibody light chains (LCs) on contact hypersensitivity (CHS) reaction. CHS reaction was measured as ear swelling response in either (**a**) recipients of wild type (WT), TNP-specific CHS effector cells incubated with TNP-tolerized JH^−/−^ mouse exosomes in some instances supplemented with anti-TNP antibody LCs or heavy chains, (**b**) recipients of WT, TNP-specific CHS effector cells incubated with anti-TNP LC-supplemented exosomes from OX-tolerized JH^−/−^ mice, or (**c**) recipients of wild type (WT), either TNP or OX-specific CHS effector cells incubated with either TNP or OX-tolerized JH^−/−^ mouse exosomes that have been supplemented with either anti-TNP or anti-OX LCs. Bars express delta ± standard error (SE). *n* = 5 mice in each group. * *p* < 0.05; ** *p* < 0.01.

We have previously shown that pan immunoglobulin-deficient JH^−/−^ mouse Ts cells produce exosomes that suppress CHS effector cells by delivering miRNA-150, but after coating with hapten-specific LCs [16]. Herein we have also demonstrated that LC supplementation activates Ts cell exosomes of B cell-deficient mice for suppression. Nonetheless, it has been unconfirmed whether only LCs possess such an activity. Thus, we administered JH^−/−^ mice with haptenized MRBCs to obtain Ts cell exosomes, that we have supplemented with hapten-specific antibody LCs or heavy chains prior to treatment of adoptively transferred CHS effector cells. Remarkably, only LCs occurred to be able to restore the suppressive activity of JH^−/−^ mouse Ts cell exosomes that alone were noninhibitory (Figure 5a, group D vs. A, C and E). The above observations strongly suggested that the antigen-specificity of Ts cell exosome action is shaped by hapten-specific LCs coating their surface. To ultimately confirm this hypothesis, firstly we have isolated Ts cell exosomes from OX-MRBC-administered JH^−/−^ mice and supplemented them with anti-TNP LCs prior to treatment of TNP-specific CHS effector cells. This led to significant suppression of CHS reaction elicited with TNP hapten (Figure 5b, group D vs. A). Furthermore, we have isolated Ts cell exosomes from TNP-MRBC-administered JH^−/−^ mice and supplemented them with anti-OX LCs prior to treatment of TNP-specific CHS effector cells, which failed to suppress elicited CHS reaction, while anti-TNP LC-coated exosomes were strongly suppressive (Figure 5c, group D vs. A and C). Analogously, Ts cell exosomes of OX-MRBC-administered JH^−/−^ mice, when supplemented with anti-TNP LCs, failed to inhibit adoptively transferred OX-specific CHS effector cells, while anti-OX LC-coated exosomes were suppressive (Figure 5c, group H vs. E and G). These findings unequivocally proved that hapten-specific antibody LCs confer antigen-specificity of Ts cell exosome-mediated suppression of CHS effector cells. 

## 3. Discussion

Hapten-specific LCs, secreted by B1 cells activated at the time of contact sensitization, are well recognized agents involved in initiation of the effector phase of CHS reaction [6]. They mostly coat the membrane of mast cells to then bind the haptenic antigen, which as a consequence induces mast cell release of proinflammatory and vasoactive mediators and initiates the inflammatory reaction. This is crucial to enable the migration of CHS effector T cells to the site of hapten deposition [23]. Further, antigen-specific LC-mediated activation of mast cells is also observed in various allergic [24] and chronic inflammatory disorders as an alternate pathway to surface receptor-bound IgE cross-linking [4]. The proinflammatory activity of LCs was recently considered in pathogenesis of different inflammation-related diseases, like inflammatory bowel disease [25], as well as lung [26] and renal disorders [25]. LCs were found in urinary exosome-like nanovesicles collected from patients with light chain amyloidosis [27]. Freely circulating LCs are also suspected to induce tissue injury in multiple myeloma patients and they could be contained in serum extracellular vesicles to support their docking to endothelial and myocardial cells [28]. On the other hand, very little is known about the possible beneficial immune regulatory role of antigen-specific LCs. Our current research findings demonstrated that hapten-specific LCs confer the antigen specificity of immune tolerance mechanism mediated by miRNA-150-carrying exosomes. To our best knowledge, this is the first demonstration of the role of LCs in cell-to-cell communication via exosomes, which ensures the specificity and selectivity of cell targeting by exosome carriers. This in turn provides the highly specific pathway of delivery of exosome cargo to the desired acceptor cell [19,29].

**Figure 6:**
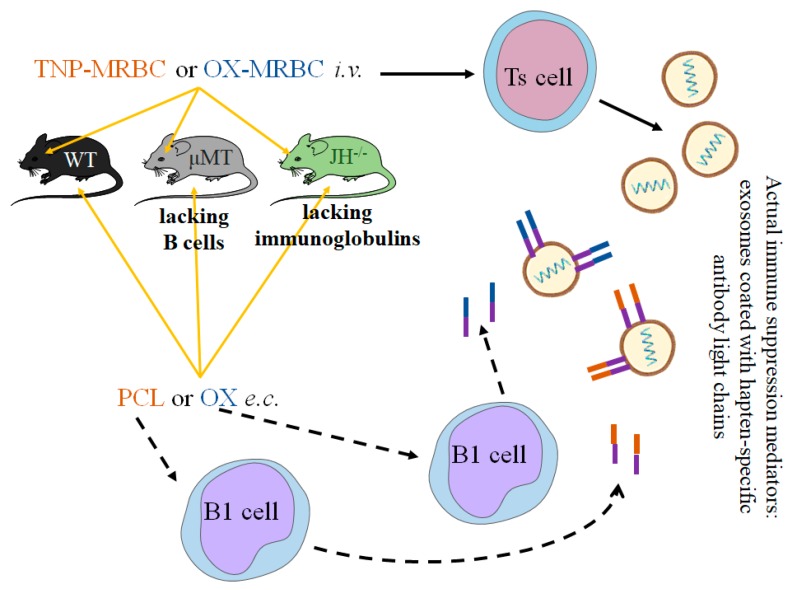
Summary figure depicting the role of hapten-specific antibody light chains secreted by B1 cells in immune suppression mechanism mediated by suppressor T (Ts) cell-derived exosomes. Epicutaneous (e.c.) sensitization of wild type (WT) mice with picryl chloride (PCL, trinitrophenol chloride) or oxazolone (OX) activates B1 cells to release hapten-specific antibody light chains that coat suppressive exosomes, which ensures hapten-specificity of exosome-mediated immune regulatory mechanism. This was proved by employing B-cell-deficient μMT and pan immunoglobulin-deficient JH^−/−^ mice, which Ts cells, after tolerization with hapten-coupled syngeneic mouse red blood cells (MRBC), produced exosomes that were nonsuppressive, unless in vitro supplemented with antibody light chains.

Our results (Figure 6) showed that hapten-specific LCs coating Ts cell-derived exosomes are secreted by B1 cells, activated by NKT cells at the time of skin sensitization with hapten [6,7,8,9,10,11]. Hence, B1 cells are involved not only in activation [20], but likely also in regulation of CHS reaction. Further, our results provided the first demonstration of the downregulatory function of hapten-specific LCs during the course of CHS reaction, executed by complementing suppressive exosomes with antigen-recognition system. It is worth noting that multiple adjacent LCs coated on the exosome surface likely gain great avidity, enabling the strictly specific binding of the antigen [19]. Consequently, this would allow exosomes to target those antigen-presenting cells that express the corresponding antigenic determinant complexed with MHC, as is suspected in the case of Ts cell exosomes [18,19]. Recently exosomes are considered a promising natural nanocarriers of genetically encoded information and biologically active compounds for various therapeutic applications [19,29]. Thus, such a strict specificity of antigen binding by LC-coated exosome carriers, addressing them to selected cells, has great clinical implications.

Our current observations confirmed that administration of altered-self MRBCs is sufficient to stimulate Ts cells to release suppressive exosomes. However, they become functionally active after coating with LCs of specificity dictated by sensitizing antigen. This has a great clinical importance, implying that alteration of self-red blood cells may induce the tolerant state conferred by Ts cell exosomes, whose suppressive action would be initiated by coating with antigen-specific LCs delivering an information about an invading antigen. The beneficial effect would result from the possibility to transiently activate the tolerance to nonpathogenic antigens, like allergens, self-antigens, or antigens of transplanted organs. On the other hand, however, this mechanism might also be involved in the unwanted immune tolerance that may develop during chronic infections.

The process of coating Ts cell exosomes by hapten-specific LCs remains uncharacterized and requires further studies. However, our preliminary results suggest the involvement of exosome membrane phospholipids [19]. Along these lines, saturated phosphocholine lipids in monocyte membranes were shown to bind LCs that were suggested to support the process of antigen capturing and engulfment [30]. In addition, LC aggregates coupled to phospholipids were found on myeloma plasma cells [31].

Extracellular vesicles, exosomes especially, are considered very promising therapeutic delivery tools and biomarkers of various diseases [29,32,33,34,35,36]. Accordingly, liposomes coated with monoclonal antibodies were designed for clinical application to specifically deliver therapeutic agents [37]. Data reported here and previously [16,17] defined a similar mechanism involving exosomes and LCs that occurs physiologically during the process of immune response regulation. In addition, lipids, supposedly, are also binding sites for LCs onto exosome membrane. It is worth noting that physiologically, in vivo produced exosomes express much greater potential as natural therapeutic agents and nanocarriers than synthetically engineered liposomes [38]. Thus, liposomes seem not to be able to fully substitute exosomes.

Among other extracellular vesicles, exosomes were found to carry various bioactive compounds, including nucleic acids, proteins, and lipids that may be immunogenic or express immune regulatory properties [39], thus, constituting cell–cell communication conveyors of great interest and numerous potential applications [40]. To deliver their cargo, exosomes in most cases fuse with targeted cells [41]. However, intensive studies have been undertaken to assess whether exosomes target the eventual acceptor cell selectively and specifically. Our current results bring novel important discovery in this regard by demonstrating that exosomes could be surface coated with hapten-specific LCs, which allows to execute the exosome function in an antigen-specific manner by targeting particular cell population. Thus, specific LCs that coat exosomes dictate the specificity of cell targeting. Accordingly, the manipulable specificity of cell targeting by exosomes would allow for the delivery of selected cargo to desired acceptor cells, and specific LCs seem to be promising candidates to ensure such a possibility for further therapeutic utilization.

To summarize, current research expands our understanding of the newly discovered tolerance mechanism mediated by Ts cell exosomes carrying miRNA-150 [15,16,17,18,19] by uncovering the function of antigen-specific LCs in exosome-mediated cell–cell communication that express great translational potential in designing nanocarriers for specific targeting of desired cells.

## 4. Materials and Methods

### 4.1. Mice

Eight to 12-week old mice of the following inbred strains: BALB/c, C57BL/6, pan immunoglobulin-deficient JH^−/−^ (BALB/c background) [16], and B cell-deficient µMT mice (C57BL/6 background) [20] or NKT cell-deficient Jα18^−/−^ mice (C57BL/6 background) [22] were from Jackson Laboratories (Bar Harbor, ME, USA) and CBA mice were from the Breeding Unit of the Jagiellonian University Medical College, Faculty of Medicine (Krakow, Poland). Mice were kept in standard conditions and fed autoclaved food and water ad libitum.

### 4.2. Ethics Statement

This study was carried out in accordance with the principles of the Basel Declaration. All experiments were approved by the Institutional Animal Care and Use Committee of the Yale University, New Haven, CT, USA (Permit Number 07381) and First Local Ethics Committee of the Jagiellonian University, Krakow, Poland (Permit Number 40/2011 and 106/2012). All procedures were performed under ether or isoflurane anesthesia, and all efforts were made to minimize suffering.

### 4.3. Coupling of Mouse Red Blood Cells with Hapten or Protein Antigen

Syngeneic MRBCs isolated from peripheral blood collected on anticoagulant (ethylenediaminetetraacetic acid, EDTA) were chemically coupled with TNP hapten by 20 min incubation on roller, at room temperature, and in darkness with 2,4,6-trinitrobenzene sulfonic acid (TNBSA, Eastman Chemicals, Rochester, New York, NY, USA) dissolved in cacodylic buffer in a ratio of 40 mg of TNBSA per 7 mL of solution per 1 mL of 100% MRBCs [14,16]. Afterwards, TNP-coupled MRBCs (TNP-MRBCs) were extensively washed with DPBS. OX hapten (Sigma, St. Louis, MO, USA) for MRBC coupling was prepared by dissolving in 96% ethanol in a ratio of 10 mg per mL (*w*/*v*) and rapid mixing with hot DPBS on roller. Then, 10 mL of 10% DPBS suspension of MRBCs was mixed with 20 mL of OX solution and incubated for 10 min on roller, at room temperature and in darkness [16]. Finally, OX-coupled MRBCs (OX-MRBCs) were extensively washed with DPBS. MRBCs were coupled with BSA by 60-min incubation on roller, at room temperature, of 1 mL of 50% MRBCs suspension in DPBS with 2.5 mL of 1% BSA solution in DPBS in the presence of 1-ethyl-3-(3-dimethylaminopropyl)carbodiimide (EDC, Pierce, Thermo Fisher Scientific, Waltham, MA, USA), used as coupling facilitating agents. At the end, BSA-coupled MRBCs (BSA-MRBCs) were washed with DPBS.

### 4.4. Induction of Tolerance by Intravenous Administration of Antigen-Coupled Syngeneic MRBCs Prior to Elicitation of CHS Reaction

Mice were intravenously injected on day 0 and 4 with 0.25 mL of nylon-filtered, 10% DPBS suspension of either TNP-MRBCs, OX-MRBCs, or BSA-MRBCs. After next 3 days mice were actively contact sensitized with either picryl chloride (PCL, trinitrophenyl chloride, Chemtronix, Swannanoa, NC, USA) or OX by application of 0.15 mL of 5% PCL or 3% OX solution, respectively, in an ethanol:acetone mixture (3:1 *v*/*v*) on shaved abdominal skin. Five days later mice were challenged to elicit CHS by topical application of 10 µL of, respectively, 0.4% PCL or 0.4% OX in acetone:olive oil mixture (1:1 *v*/*v*) on each side of both ears. After 24 h ear swelling was measured with an engineer’s micrometer (Mitutoyo, Takatsu-ku, Kawasaki, Kanagawa, Japan) by a blinded observer [16,17,18,42]. Background ear thickness, measured before challenge, was subtracted to yield a value of ear thickness increase for each mouse. Nonspecific increase in ear thickness in nonsensitized, but similarly challenged littermate animals, resulting from chemical skin irritation by hapten and its vehicle, was subtracted from experimental groups to yield a net swelling value expressed as delta ± standard error (SE) [U × 10^−2^ mm].

### 4.5. Isolation of Suppressive Exosomes Generated by Suppressor T Cells of Tolerized Mice and Adoptive Transfer of CHS Effector Cells

Mice were injected twice with TNP or OX-coupled MRBCs and 3 days later epicutaneously sensitized with PCL or OX solutions, as described above. Two days later lymph nodes and spleens were collected for culture of their single cell suspensions (2 × 10^7^ cells per mL) in protein-free Mishell–Dutton medium [16,17,18] lasting for 48 h at 37 °C and 5% CO_2_. The resulting culture supernatant was subsequently centrifuged at 300× *g* and 3000× *g* to remove cells and debris, filtered through 0.45 µm and 0.22 µm molecular filters and then ultracentrifuged twice at 100,000× *g* for 70 min at 4 °C. Pelleted exosomes from tolerized mice were resuspended in DPBS [16] and used for 30-min incubation in 37 °C water-bath with lymph node and spleen CHS effector cells collected from mice sensitized with PCL or OX (see above) [16,17,18]. After washing, CHS effector cells were transferred intravenously (7 × 10^7^ cells per mouse) into naive recipients that were immediately challenged with PCL or OX to elicit CHS response, as described above. The exact molecular characteristics of exosomes, confirmed within the current study (Figure S1), along with confirmation of their Ts cell origin, was recently reported [16,17]. Where indicated, exosomes were incubated overnight on ice with anti-hapten (TNP or OX) monoclonal antibody kappa LCs [16], or heavy chains, both produced as formerly described [43], followed by ultracentrifugation to remove unbound LCs or heavy chains. Control, nonsuppressive nanovesicles were obtained from ultracentrifuged supernatants of 48-h cultures of lymph node and spleen cells from naive mice [16,18].

### 4.6. Data Analysis

Each experiment was carried out 2–3 times and the results of representative experiments were shown in the figures. All groups consisted of five mice. Statistical significance of the data was estimated (after control of meeting of test assumptions) in one-way Analysis of Variance (ANOVA) with post hoc RIR Tukey test and *p* < 0.05 was considered statistically significant.

## Supplementary Materials

Supplementary materials can be found at http://www.mdpi.com/1422-0067/19/9/2656/s1.

## Abbreviations

BSA	Bovine serum albumin
BSA-MRBCs	Bovine serum albumin-coupled mouse red blood cells
CHS	Contact hypersensitivity
LCs	Light chains
MRBCs	Mouse red blood cells
OX	Oxazolone
OX-MRBCs	Oxazolone-coupled mouse red blood cells
PCL	Picryl chloride (trinitrophenol chloride)
TNBSA	2,4,6-trinitrobenzene sulfonic acid
TNP	2,4,6-trinitrophenol
TNP-MRBCs	trinitrophenol-coupled mouse red blood cells
Ts	T suppressor (cells)
WT	Wild type
