# Supplementary figures and images for: Antibody Light Chains Dictate the Specificity of Contact Hypersensitivity Effector Cell Suppression Mediated by Exosomes
Source: Int J Mol Sci. 2018 Sep 7;19(9):2656. doi: 10.3390/ijms19092656 (PMC6163184; doi:10.3390/ijms19092656)

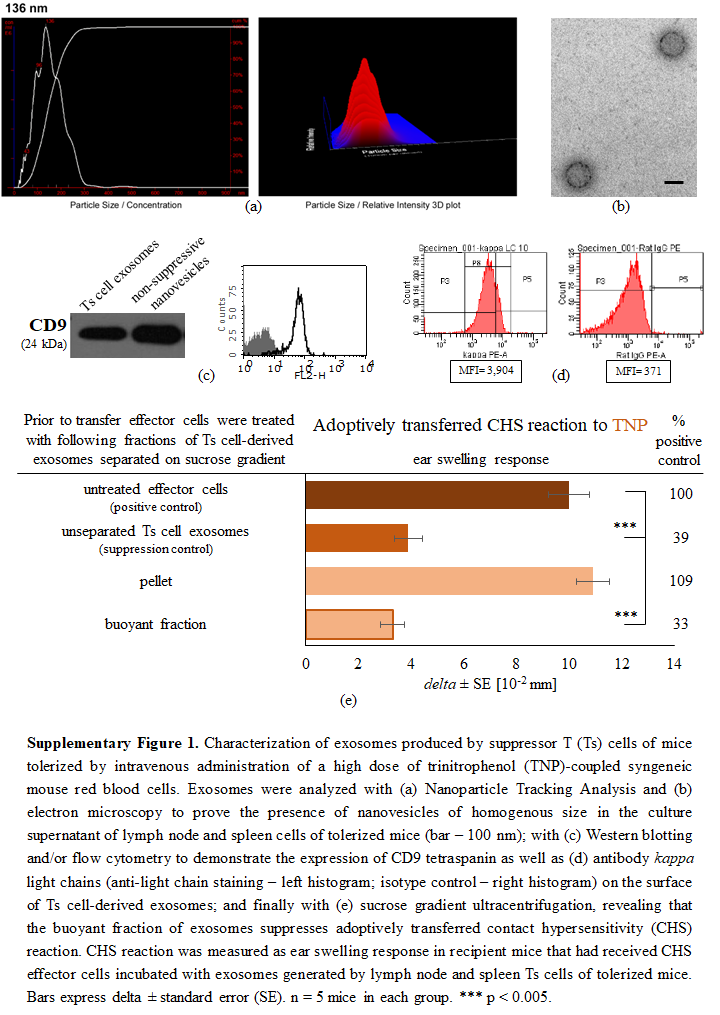

Supplement: Supplementary file 1 [file ijms-19-02656-s001.zip › ijms-345305-supplementary.tif]
